# Subspecialized radiology reporting: productivity and impact on the turnaround times for radiology reports in a middle-income country

**DOI:** 10.1590/0100-3984.2019.0089

**Published:** 2020

**Authors:** Nupur Verma, Gabriel Sartori Pacini, Juliana Pastorino Torrada, Diogo Martins de Oliveira, Matheus Zanon, Edson Marchiori, Tan-Lucien Mohammed, Bruno Hochhegger

**Affiliations:** 1 Department of Radiology, College of Medicine, University of Florida, Gainesville, FL, USA.; 2 Medical Imaging Research Lab (Labimed), Department of Radiology, Pavilhão Pereira Filho Hospital, Irmandade Santa Casa de Misericórdia de Porto Alegre, Porto Alegre, RS, Brazil.; 3 School of Medicine, Graduate Program in Medicine and Health Sciences, Pontifical Catholic University of Rio Grande do Sul, Porto Alegre, RS, Brazil.; 4 Federal University of Rio de Janeiro (UFRJ), Rio de Janeiro, RJ, Brazil.

**Keywords:** Radiologists/classification, Medical records/standards, Radiology department, hospital/organization & administration, Radiology information systems/standards, Radiology/organization & administration, Specialization/trends, Radiologistas/classificação, Registros médicos/normas, Serviço hospitalar de radiologia/organização & administração, Sistemas de informação em radiologia/normas, Radiologia/organização & administração, Especialização/tendências

## Abstract

**Objective:**

To evaluate the effect that transitioning from a model of general radiology reporting to one of subspecialized radiology reporting has on report turnaround times (TATs) and on productivity in the radiology department of a hospital in a middle-income country.

**Materials and Methods:**

The reporting workflow in our radiology department was changed from general reporting (any radiologist reporting imaging studies for any specialty) to subspecialized reporting (radiologists exclusively reporting imaging studies that fall within their subspecialty-abdominal, musculoskeletal, cardiothoracic, emergency, or neurological imaging). This was a retrospective study in which we compared general reporting with subspecialized reporting in terms of the following variables: the TAT; the proportions of reports completed within 2 h and within 24 h (TAT-2h and TAT- 24 h, respectively); and productivity. Data were collected over two 24-month periods (2015-2016 for general reporting and 2017-2018 for subspecialized reporting).

**Results:**

A total of 208,516 reports were generated. The median report TAT decreased from 49.1 h and 52.9 h in 2015 and 2016, respectively, to 16.1 h and 15.2 h in 2017 and 2018, respectively (*p* < 0.001). The TAT-2h also improved, increasing from 8.7% and 7.9% in 2015 and 2016, respectively, to 52.0% and 61.3% in 2017 and 2018, respectively (*p* < 0.001), as did the TAT- 24 h, which increased from 12.1% and 14.1% in 2015 and 2016, respectively, to 74.3% and 78.7% in 2017 and 2018, respectively (*p* < 0.001). Between the two periods, the total number of scans performed increased by 33% (*p* = 0.001).

**Conclusion:**

The implementation of a subspecialized reporting system significantly improved the median TAT for radiology reports, as well as increasing the TAT-2h and TAT- 24 h, during a time of increased productivity.

## Abstract (pt)

**Objetivo:**

Avaliar o efeito da transição de um modelo de laudos em radiologia realizados por radiologistas gerais para um modelo relatado por subespecialização no tempo de resposta de relatórios (TAT) em radiologia e produtividade em um departamento de radiologia em um país em desenvolvimento.

**Materiais e Métodos:**

O fluxo em nosso departamento foi modificado de um sistema de relatórios gerais (qualquer radiologista lauda exames de imagem de qualquer especialidade) para um sistema de subespecialização (radiologistas laudam exclusivamente exames de imagem pertencente a sua subespecialidade - abdominal, musculoesquelético, cardiotorácico, emergência e neurologia). Este estudo retrospectivo avaliou a TAT, TAT-2 horas e TAT-24 horas e a produtividade num período de 24 meses de relatórios gerais (2015-2016) comparado a um período de 24 meses do sistema de subespecialização (2017-2018).

**Resultados:**

No total, 208.516 laudos foram gerados. A TAT reportada reduziu de uma média de 49,1-52,9 horas durante o período geral para 15,2-16,1 horas durante o período de relatórios por subespecialidade (*p* < 0,001). TAT-2 horas e TAT- 24 horas aumentaram significativamente de 7,9-8,7% para 52,0-61,3% e de 12,1-14,1% para 74,3-78,7%, respectivamente (*p* < 0.001). O número total de exames aumentou em 33% (*p* = 0.001) comparando os dois períodos.

**Conclusão:**

A implementação de um sistema de laudos por subespecialidade aumentou o TAT em radiologia, incluindo benefícios no TAT-2 horas e TAT-24 horas durante um período de aumento da produtividade.

## INTRODUCTION

The exponential rise in the number of imaging examinations over the past three decades has challenged radiology departments to improve operations to achieve efficient output of reports. In the radiology department, improving the turnaround time (TAT) of radiology reports is crucial because of the increasing demand for precision and for specific examinations in all clinical settings. These growing demands on radiology services highlight the need to shorten the TAT in order to maintain a satisfactory level of productivity and to facilitate prompt patient care^(1)^. The benefits of a short TAT include an overall reduction in healthcare costs due to several factors. For inpatients, such factors include shorter hospital stays, lower costs, and faster implementation of treatment^(2)^. Previous studies have suggested that the TAT for a report of a radiology examination of an inpatient should be less than 8 h^(3)^. Shorter TATs provide similar benefits in the emergency department and in outpatient settings. More efficient production of radiology reports in outpatient settings could also lead to a decrease in hospital admissions and could improve prognoses by facilitating early diagnosis^(2,4)^.

In recent decades, the field of radiology has undergone several significant changes, including subspecialization. An American College of Radiology survey affirmed this trend, showing that 91.5% of radiology trainees intended to pursue a fellowship and that 89.9% planned to subspecialize^(5)^. Subspecialization could also improve reporting TATs and productivity, because subspecialty-trained board-certified radiologists should identify common findings, normal variants, and subspecialty pathologies faster than do general radiologists^(1,6-8)^.

Given the trend toward subspecialization, the goal of this study was to assess the impact that the shift from general to subspecialized reporting has on TATs, the proportions of radiology reports available within 2 h and within 24 h (TAT-2h and TAT- 24 h, respectively), as well as to assess the overall number of reports generated at a hospital in a middle-income country.

## MATERIALS AND METHODS

### Study design

We retrospectively evaluated imaging reports issued between January 2015 and December 2018. Reports acquired outside core work hours were excluded. If a finalized report was submitted to revision, the TAT for that report was based on the date and time of the first final signature before the revision.

This study was approved by our institutional review board. Because of the retrospective nature of the study, the requirement for written informed consent was waived. All study procedures were in accordance with the ethical standards of the institutional research committee, as well as with those outlined in the 1964 Helsinki declaration and its later amendments.

### Workflow

Prior to the initiation of this investigation, our institution successfully implemented the integrated Radiology Information System/Picture Archiving and Communication System (MV Informática, Fortaleza, Brazil) solution, as well as voice recognition software SpeechMagic SDK (Nuance Communications, Dublin, Ireland), so that all of the radiologists had a suitable amount of time to familiarize themselves with the workflow. Residents are not authorized to sign reports. Therefore, preliminary reports prepared by residents were not used in order to calculate the TAT if a board-certified radiologist had not signed the report.

### Outcome measures

Sources in the literature have defined the time to the “reported” status of a radiology examination as “the time from the completion of image acquisition to the availability of the final radiology report”^(7)^. The TATs were drawn from the Radiology Information System, through the use of a calculation tool integrated into the Cockpit software (MV Informática). The TAT- 24 h was determined, and the TAT for each report was calculated on the basis of the time and date of the final signature by a board-certified radiologist.

To measure productivity, we determined the monthly number of radiology reports generated per radiologist. The radiologists included were full-time (1.0 full-time equivalent) physicians. Radiologists who had less than 1.0 full-time equivalent activities were excluded. We calculated the immediate and cumulative proportions of full- and part-time radiologists. We excluded radiologists with absences of four or more successive weeks. Only board-certified radiologists were included in the full-time radiologist group. During the study period, there was no extra compensation to radiologists linked to their productivity, which could have introduced an external bias in the analyses of TATs and radiologist performance.

The overall TAT, TAT- 2 h, and TAT- 24 h were analyzed on the basis of the time and date of the final signature of a report by a board-certified radiologist. Productivity was analyzed by determining the number of reports issued monthly by full-time radiologists between January 2015 and December 2018. During the study period, neither the number of hours of work nor shift length changed for any of the radiologists included.

### Reorganization from general to subspecialized reporting

Our radiology department was initially composed of radiology physicians and residents who interpreted all imaging modalities from a common worklist. In that model, radiologists selected examinations as they wished, regardless of their subspecialty training (or lack thereof). In January 2017, our institution established a system of subspecialized reporting for the following imaging subspecialties: abdominal, musculoskeletal, cardiothoracic, emergency, and neurological. The radiologists were grouped by subspecialty in order to replace general reporting in the current organization by the end of 2016. The implementation period was from July 2016 to January 2017.

The grouping by subspecialty was performed according to certain criteria. Radiologists who had completed at least 6 months of specific fellowship training were grouped by fellowship specialty. Such radiologists accounted for 17 of the 22 radiologists at our institution. Those who had not undertaken a fellowship^(5)^ were asked to choose a field in which they felt most competent and confident based on their comfort level in practice. Those radiologists were offered additional training by joint reading, as well as specialty workshops on topics such as coronary computed tomography angiography.

### Statistical analysis

Data distribution was assessed with the Shapiro-Wilk test. Two-tailed *p*-values less than 0.05 were considered statistically significant. Data with a non-normal distribution are presented as medians and interquartile ranges. Variables were compared by using the Mann-Whitney U-test.

The TAT- 24 h/TAT- 2 h ratio was calculated with Pearson chi-square tests and quantified with odds ratios. The impact of the TAT- 24 h was determined by logistic regression with the generalized additive model package^(7)^. The report TAT was calculated on the basis of the time point at which the report was finalized by a board-certified radiologist. The TAT- 2 h and TAT- 24 h were compared by using the Mann-Whitney U-test. All statistical analyses were performed with the IBM SPSS Statistics software package, version 22 (IBM Corp., Armonk, NY, USA).

## RESULTS

Among the 22 radiologists evaluated, the distribution by subspecialty was as follows: abdominal imaging (n = 7); musculoskeletal imaging (n = 2); cardiothoracic imaging (n = 5); emergency imaging (n = 5); and neurological imaging (n = 3). During the study period, 208,516 radiology reports were generated at our institution: 92,130 (44.18%) in the 2015-2016 period and 116,386 (55.82%) in the 2017-2018 period. That corresponds to a 26.32% increase in the number of reports after the implementation of the subspecialized reporting system. Production per year is presented in Table 1.

**Table 1:** Numbers of scans and report TATs.

					TAT-2h		TAT-24h		TAT*
Year	OS-CT	CT	MRI	CT/MRI	n	(%)	n	(%)	h
2015	16,234	32,511	13,092	45,603	3967	(8.7)	678	(12.1)	49.1
2016	128,888	33,388	13,139	46,527	3676	(7.9)	6560	(14.1)	52.9
2017	23,133	43,041	13,656	55,697	28,962	(52.0)	41,383	(74.3)	16.1
2018	28,204	45,758	14,931	60,689	37,202	(61.3)	47,762	(78.7)	15.2

OS-CT, out-of-system CT scans (those requested by physicians outside of our institution); TAT-2h, reports available within 2 h; TAT-24h, reports available within 24 h.

* OS-CT scans were excluded from the calculations.

**Figure 1:**
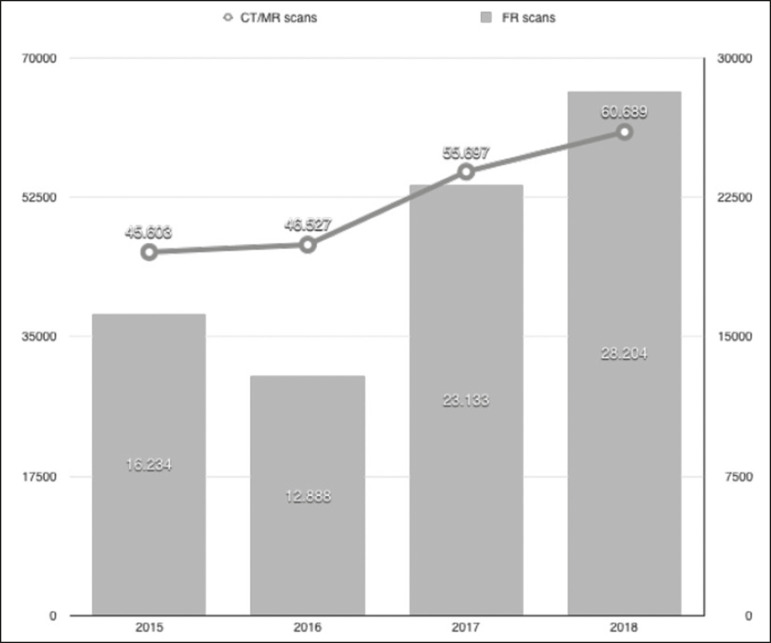
Number of imaging examinations per year, 2015-2018. Quantitative graphic showing the number of scans performed at our facility during the study period. Note the substantial increase in the use of all imaging modalities in the 2017-2018 period (after implementation of a subspecialized radiology reporting system) in comparison with the 2015-2016 period (under the general radiology reporting system).

When comparing the 2015-2016 period with the 2017-2018 period, the numbers of magnetic resonance imaging (MRI) scans, computed tomography (CT) scans, and out-of-system CT scans (those requested by physicians outside of our institution) increased by 14.04% (from 13,092 to 14,931), 40.74% (from 32,511 to 45,758), and 73.74% (from 16,234 to 28,204), respectively (Figure 1). Collectively, the number of CT and MRI scans increased by 33.08% (from 45,603 to 60,689).

**Figure 2:**
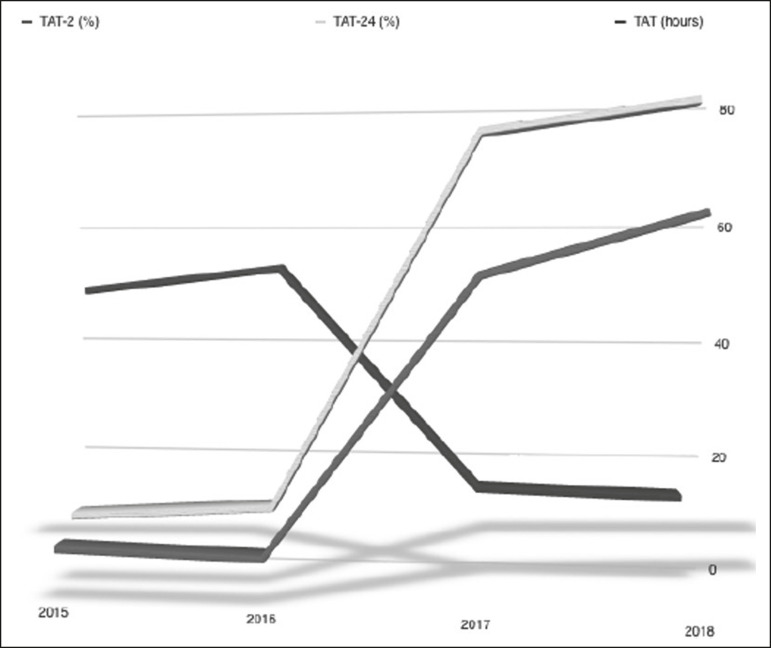
Proportion of TAT-2h, TAT-24h, and overall TAT (in h) for radiology reports of all imaging examinations performed during the period of general reporting (January 2015 to December 2016) and during the period of subspecialized reporting (January 2017 to December 2016).

Median report TAT decreased from 49.1 h and 52.9 h in 2015 and 2016, respectively, to 16.1 h and 15.2 h in 2017 and 2018, respectively (*p* < 0.001). After implementation of the subspecialized reporting system, there were also significant improvements in the TAT- 2 h-from 8.7 % and 7.9% in 2015 and 2016, respectively, to 52.0% and 61.3% in 2017 and 2018, respectively (*p* < 0.001)-and in the TAT- 24 h-from 12.1% and 14.1% in 2015 and 2016, respectively, to 74.3% and 78.7% in 2017 and 2018, respectively (*p* < .0001)-as can be seen in Figure 2. Neurological and cardiothoracic imaging presented the best improvement in the overall TAT, with increases of 68% and 53%, respectively.

## DISCUSSION

In this study, we analyzed the change from a general to a subspecialized radiology reporting system and its impact on report TATs. The new system improved the TATs of radiology reports and radiologist productivity, as well as increasing the proportions of radiology reports available within 2 h and within 24 h. We demonstrated that changing from a general to a subspecialized reporting system significantly decreased the median report TAT by 69.04%.

Our results are in line with those of previous reports^(1,8)^. In a study conducted by Stern et al.^(1)^, a change from general to subspecialized radiology reporting resulted in a significant decrease in the median TAT, which dropped from 17.04 h to 3.38 h. In another study, subspecialized reporting led to an increase in productivity of approximately 7.0% at one year after implementation^(8)^. In comparison, we found a 19.70% increase in productively after one year of subspecialized reporting. In the present study, TATs for all imaging modalities were reduced substantially after the implementation of subspecialized reporting.

The change from general to subspecialized reporting occurred in parallel with a period of significant increases in per-radiologist productivity and in the overall number of radiology reports. We believe that those increases were due to several factors. Compared with general reporting, subspecialized reporting is likely more efficient in terms of time management, as evidenced by the shorter report TATs. However, the increased volume of reports could also be attributed to external factors such as an increase in the number of imaging examinations ordered by physicians in all clinical settings. It is essential to note that this increase in productivity was not related to longer working hours or to an increase in the number of shifts worked per radiologist.

Our study has some limitations. First, it was a single-center study conducted at a hospital with resident training. Therefore, it might not be possible to generalize our results to centers without training programs or with different program sizes and resident levels (e.g., fellows vs. junior residents). It is vital to balance the goal of reducing TATs with that of preserving the educational exposure of trainees to cases. Report TATs are probably shorter at private imaging centers, where each examination is completed on a first-look basis, than at training centers, where the most common model is resident review followed by a read-out session. In addition, teaching hospitals like ours tend to receive a greater proportion of high-complexity cases, which require more time to report and consequently increase the report TATs. The benefit of a subspecialized radiology reporting system may also be more pronounced at our center, where complex postoperative imaging examinations, which require familiarity not only with the expected findings but also with the common complications, are commonplace. Furthermore, the improvement in the TATs for subspecialties could be explained by the informal knowledge about cases that subspecialized radiologists have. For example, if an abdominal radiologist has prior exposure to a surgical approach, surgical anatomy, or treatment trends from attending multidisciplinary conferences, that radiologist might spend less time in reporting than would a general radiologist, who might need to perform an extensive review of chart notes. We must also consider that our comparison of general and subspecialization organization at different times in our radiology practice might have resulted in an underestimation of the influence of other changes, such as those in the clinical demands of the hospital, in the expectations of the clinicians in terms of what is considered an acceptable TAT, and even in the preference of requesting physicians in terms of the number of reads. Influences by individual radiologists (e.g., that of radiologists who read out examinations one at a time with trainees and that of those who batch read at specific periods) are also difficult to account for. Such factors provide additional opportunities for reducing TATs in radiology reporting.

## CONCLUSION

The implementation of a subspecialized reporting system significantly improved the median TAT for radiology reports, including improvements in the TAT- 2h and TAT- 24 h, during a time of increased productivity at a hospital in a middle-income country. The benefit of a subspecialized model is that it can help avoid unnecessary hospitalizations, shorten hospital stays, and reduce costs, especially in the context of the increasing demand for radiology examinations.
